# Targeting the motor regulator Klar to lipid droplets

**DOI:** 10.1186/1471-2121-12-9

**Published:** 2011-02-24

**Authors:** Yanxun V Yu, Zhihuan Li, Nicholas P Rizzo, Jenifer Einstein, Michael A Welte

**Affiliations:** 1 Department of Biology, University of Rochester, Rochester, NY 14627, USA; 2 University of Massachusetts Dartmouth, 285 Old Westport Road, North Dartmouth, MA 02727-2300, USA

## Abstract

**Background:**

In *Drosophila*, the transport regulator Klar displays tissue-specific localization: In photoreceptors, it is abundant on the nuclear envelope; in early embryos, it is absent from nuclei, but instead present on lipid droplets. Differential targeting of Klar appears to be due to isoform variation. Droplet targeting, in particular, has been suggested to occur via a variant C-terminal region, the LD domain. Although the LD domain is necessary and sufficient for droplet targeting in cultured cells, lack of specific reagents had made it previously impossible to analyze its role *in vivo*.

**Results:**

Here we describe a new mutant allele of *klar *with a lesion specifically in the LD domain; this lesion abolishes both droplet localization of Klar and the ability of Klar to regulate droplet motion. It does not disrupt Klar's function for nuclear migration in photoreceptors. Using a GFP-LD fusion, we show that the LD domain is not only necessary but also sufficient for droplet targeting *in vivo*; it mediates droplet targeting in embryos, in ovaries, and in a number of somatic tissues.

**Conclusions:**

Our analysis demonstrates that droplet targeting of Klar occurs via a cis-acting sequence and generates a new tool for monitoring lipid droplets in living tissues of *Drosophila*.

## Background

Lipid droplets are the intracellular sites for storage of neutral lipids. Long dismissed as inert inclusions, they are now recognized as dynamic organelles with a myriad of functions well beyond fat storage [reviewed in, [1], [2]]. In many cells, lipid droplets are highly motile, actively moving along cytoskeletal tracks [3]. Such motion is implicated in the delivery of nutrients [4,5], the growth and turnover of lipid droplets [6-8], the exchange of lipids and proteins between various cellular compartments [9-12], and even the assembly of viral particles [13].

Despite the ubiquity and potential biological significance of droplet motion, its mechanism is not well understood [3]. Most characterized droplet motion occurs along microtubules, driven by motor proteins, such as the minus-end directed cytoplasmic dynein [8,13-16] and the plus-end directed kinesin-1 [17]. The same motor proteins are also employed in many other transport processes and are responsible for the motion of various vesicles, mitochondria, RNP particles, chromosomes and nuclei. Motion of these cargoes is regulated distinctly from that of lipid droplets [*e.g*., [18,19]].

At least in part, specificity of droplet motion is achieved via distinct motor regulators that are present exclusively on lipid droplets. For example, in both mammals and flies, members of the Perilipin family modulate droplet motion [6,13,20]; these proteins localize largely or exclusively to lipid droplets [21]. In *Drosophila *embryos, droplet motion is controlled by Klarsicht (Klar) [19], a protein highly enriched on lipid droplets at this stage of development [22]. Presumably, unique motor regulators present only on droplets control specifically those motors attached to this cargo.

Klar's role is not limited to lipid droplets; in different cells, Klar controls distinct transport processes. In early embryos, it regulates the bidirectional motion of lipid droplets [19], which are transported by cytoplasmic dynein [14] and kinesin-1 [17]. In embryonic salivary glands, in contrast, Klar is required for efficient transport of secretory vesicles [23], possibly by modulating the activity of cytoplasmic dynein. In developing photoreceptors, Klar promotes apical migration of nuclei [24]. Motion of these nuclei is powered by cytoplasmic dynein [25], and also involves the activity of kinesin-1 [26]. The intracellular localization of Klar reflects these varying functions in different tissues: in early embryos, Klar is present on lipid droplets [22]; in photoreceptors, it is enriched on the nuclear envelope [24,27]. Apparently, Klar regulates the same or a similar set of motors in all these instances, and it is the location of Klar that determines which subset of cellular motors is controlled. Thus, determining how Klar is targeted intracellularly is a key to understanding its cargo specificity.

This tissue-specific targeting of Klar correlates with isoform variation [22]. The *klar *locus encodes at least three protein isoforms that are due to the use of multiple promoters and Poly A sites [22]. The alpha (α) isoform is required for nuclear migration in photoreceptors [22,24]; the beta (β) isoform controls the motion of embryonic lipid droplets [22]; no function has yet been described for the gamma (γ) isoform. Because the α and β isoforms are predicted to have identical N-terminal regions of 1726 amino acids, but carry unique C-terminal regions (536 aa for Klar α, 114 aa for Klar β), these C-terminal domains may be cis-acting targeting sequences that dictate where in the cell Klar accumulates [22].

For Klar α, there is ample support for this model. Its C-terminal region contains a 60 aa KASH (Klarsicht/ANC-1/Syne Homology) domain. KASH domains typically localize to the outer nuclear envelope, and together with SUN domain proteins in the inner nuclear envelope establish a bridge linking cytoplasmic and nucleoplasmic proteins [28,29]. Indeed, the KASH domain of Klar is sufficient to target unrelated proteins to the nuclear envelope, in cultured cells [22] as well as in photoreceptors [30]. In addition, mutations of Klar α that truncate the protein within or just N-terminal to the KASH domain fail to localize to the nuclear envelope [22,30]. Thus, the KASH domain is necessary and sufficient to target Klar to the nuclear envelope.

Available evidence is consistent with the notion that the unique C-terminal region of Klar β has an analogous role in targeting to lipid droplets. For example, in cultured cells, this so-called LD domain is sufficient to recruit RFP to lipid droplets [22]. However, whether the LD domain is necessary and sufficient for droplet targeting *in vivo *has not been clearly established. For one, the exact distribution of LD fusion proteins in cultured cells is distinct from the distribution of endogenous Klar *in vivo*: in embryos, Klar β is present in one or a few dots per lipid droplet, suggesting that it may be recruited to privileged sites [22]. In cultured cells, in contrast, RFP-LD fusions are evenly distributed all over the droplet surface, raising the possibility that targeting in the two systems occurs by distinct mechanisms. From mutational analysis, it is clear that *in vivo *droplet localization requires C-terminal regions of Klar β [22]. However, all available *klar *alleles either only disrupt Klar α or truncate both Klar α and Klar β within the shared N-terminal region. Therefore, they cannot be used to distinguish if droplet targeting is due to the Klar β-specific LD domain or due to regions shared between Klar α and Klar β.

To examine the role of specifically the LD domain, we identified a new *klar *allele that deletes the last 64 aa of this domain. This allele abolishes droplet localization entirely. We also expressed a GFP-LD fusion protein and find that it targets to lipid droplets in early embryos, in the female germ line, and in somatic tissues. This analysis establishes that *in vivo *the LD domain is necessary and sufficient for targeting to lipid droplets.

## Results

### Identifying a *klar *allele with a lesion in the LD domain

The transcripts for Klar α and β diverge after exon 15, followed either by exons 16, 17 and 18 (Klar α) or exon 15ext (Klar β) [22]. Exon 15ext encodes the LD domain. Klar β proteins lacking C-terminal regions fail to localize to embryonic lipid droplets [22]. All characterized *klar *alleles with lesions in Klar β remove not only the LD domain (114 amino acids), but also at least 128 amino acids encoded by exons 14 and 15, exons shared with Klar α. Thus, these reagents do not allow a conclusive test if targeting to embryonic lipid droplets requires the LD domain (unique to Klar β) or is mediated by regions common to both Klar α and Klar β.

To address this issue, we searched for new *klar *alleles with lesions specifically in the LD domain. Since Klar β null alleles are viable [22], we took advantage of a collection of EMS mutagenized, homozygously viable third chromosomes [31]. We employed TILLING (Targeting Induced Local Lesions IN Genomes) [32-34] to find sequence changes in a genomic region that includes *klar *exon 15ext. We recovered two candidate lines (Z3-3772, Z3-1711) with predicted sequence changes in exon 15ext. We focused on the potential *klar *mutation in line Z3-3772 since it was predicted to result in deletion of roughly half of the LD domain.

**Figure 1:**
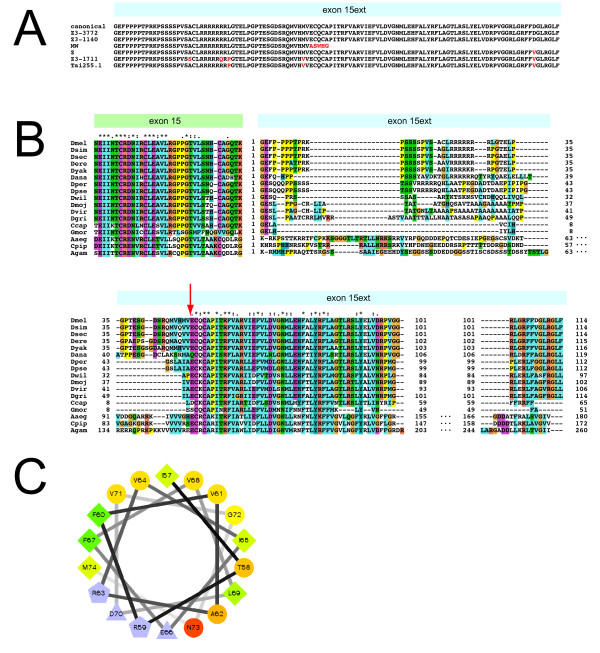
**Sequence of the LD domain in various strains and species**. (A) Comparison of *D. melanogaster *strains. Exon 15ext was sequenced in various strains, and the encoded protein was compared to the canonical sequence available on FlyBase. Shown are the *klar^MW ^*and *klar^Z ^*allele, the wild-type strain Tai255.1, as well as three mutagenized chromosomes from the Zuker collection (Z3-3772, Z3-1140, Z3-1711). Changes from the canonical sequence are indicated in red. The wild-type LD domain encompasses 114 amino acids. (B) Sequence variation in the LD domain across fly species. Candidate *klar *exons 15 and 15ext were identified in sixteen fly species based on sequence similarity to the corresponding *D. melanogaster *exons. Predicted protein sequences were aligned with Clustal2. * = position at which residues are absolutely conserved; : = position at which residues show similar chemical properties; . = partial conservation. Color scheme highlights chemically similar amino acids. A 26 aa stretch in exon 15 and a 46 aa stretch in exon 15ext are highly conserved. The red arrow indicates at which point the protein sequence of the *klar^MW ^*allele diverges from the wild-type sequence; this allele deletes the entire 46 aa conserved region (amino acids 51-96) in exon 15ext. (C) Helical wheel diagram for amino acids 57-74 of the *D. melanogaster *LD domain. Potentially charged residues are represented in light blue; for uncharged residues, hydrophobicity is indicated by a scale from red (most hydrophilic) to green (most hydrophobic). This amino-acid sequence is compatible with an amphipathic helix structure. A similar pattern of hydrophobicity is conserved across all seventeen species shown in (B).

Many lines from the mutant collection are mixtures of homozygotes and heterozygotes; the latter carry not only the mutagenized chromosome but also a balancer chromosome. In particular, for strain Z3-3772, the TILLING analysis had employed DNA from heterozygotes as in the extant stock homozygotes are rare to non-existent. When we extracted the non-balancer chromosome from this stock and sequenced exon 15ext, the predicted protein sequence of the corresponding LD domain was identical to the canonical sequence of Klar (Figure 1A).

To reconcile this observation with the TILLING results, we hypothesized that the mutation in the parental line might exist on the balancer chromosome, TM6B. We generated flies heterozygous for the TM6B chromosome from line Z3-3772 and for *Df(3L)emc^E12^*, a large deletion that removes - among many other genes - the entire *klar *locus. Genomic sequencing revealed the absence of a contiguous 25 bp stretch in the middle of exon 15ext.

This deletion is not a general feature of the TM6B balancer chromosome. Although many of the lines from the mutant collection were analyzed as heterozygotes, the deletion was recovered only once. In particular, our screen identified sequence variations for 19 other lines for which DNA from heterozygous flies had been characterized; but for none of these lines was the 25 bp deletion identified. We also extracted a TM6B chromosome from a different stock of the collection and found that the 25 bp deletion was not present. Thus, this deletion in exon 15ext did not pre-exist on the TM6B chromosome and apparently arose spontaneously since the mutagenesis scheme employed to generate the collection [31] did not expose the balancer chromosomes to EMS. In the following, we will refer to the TM6B chromosome carrying this deletion as TM6B^MW ^and to the corresponding *klar *allele as *klar^MW^*. The TM6B chromosome without this deletion and the corresponding *klar *allele will be called TM6B^Z ^and *klar^Z^*, respectively.

What is the consequence of this change in the genomic sequence? The 25 bp deletion in exon 15ext shifts the open reading frame and should result in a truncated LD domain: While the initial 50 amino acids are unchanged, the C-terminal 64 residues are replaced by an unrelated five-amino-acid sequence (Figure 1A).

To determine if *klar^MW ^*carried additional lesions, we sequenced all the coding exons of *klar *in this allele. The predicted Klar protein(s) display a number of differences to the canonical Klar sequence available on FlyBase (Table 1), but with the exception of the truncation of the LD domain, all changes are also found for allele *klar^Z^*. Furthermore, all these additional changes have been observed - individually or in combination - in presumably wild-type versions of Klar (Table 1) and thus apparently represent naturally occurring, benign variations.

**Table 1:** Predicted sequence variation in Klar^MW^

AA #	Observed change	Exon affected	Notes
633	Ser to Thr	7	a, b

1013	Ser to Thr	9	a, b

1210	Ser to Ala	11	a, c

1358	Asp to Glu	11	a, b, c

1366	Ser to His	11	a, b, c

1510	Thr to Ser	13	a, b, c

1577	Ile to Asn	14	a, b, c

Frame shift	See Fig. 1A	15ext	

1887	Thr to Ser	17	a, b

1909	Ser to Ala	17	a, b

The protein coding exons of *klar^MW ^*were sequenced, and the predicted protein sequence was compared to the wild-type Klar sequence available on FlyBase. The position of the sequence change is given relative to the protein sequence of the originally reported Klar α protein [24]. Exons numbering follows the convention established previously [22]. Notes indicate if a particular amino acid change is shared with other *klar *alleles.

^a ^present in Klar^Z^

^b ^present in a wild-type Klar α cDNA (GenBank AAD43129, as reported in [24])

^c ^present in various wild-type *klar *alleles (S. Jangi, Y. Guo, MAW, unpublished observations)

### The LD domain is necessary for droplet localization *in vivo*

The identification of *klar^MW ^*provides an opportunity to test the functional consequences of disrupting the LD domain specifically. Although the other amino acid changes encoded by *klar^MW ^*are likely benign, they could in principle be responsible for any phenotypes observed with *klar^MW^*. In the following, we therefore compare *klar^MW ^*to the *klar^Z ^*allele, which shares these changes, apart from the LD domain lesion.

**Figure 2:**
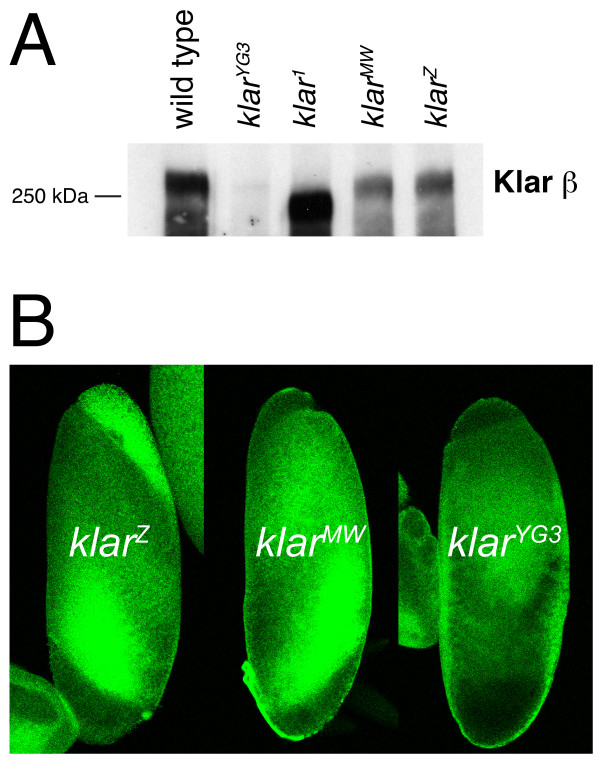
**Klar^MW ^is expressed in embryos, but not localized to lipid droplets**. (A) Klar Western of embryos (cycle 14/Phase II) of various genotypes. Since *klar^MW ^*and *klar^Z ^*are present on TM6B balancer chromosomes, these alleles were analyzed in combination with *Df(3L)emc^E12^*, a deletion that encompasses the entire *klar *locus. The Klar^MW ^protein is expressed and stable, as it accumulates to similar levels as the wild-type protein Klar^Z^. (B) Embryos were centrifuged to separate organelles by density, fixed and stained for Klar. The lipid-droplet layer was recognized by its distinctive appearance by bright-field microscopy. In this image, embryos are arranged with the lipid-droplet layer pointing up. Klar signal is enriched on the droplet layer in *klar^Z ^*embryos, but not detectable on the droplet layer when the LD domain is truncated (*klar^MW^*) or Klar β is not expressed (*klar^YG3^*).

We first asked if the mutant Klar^MW ^protein was expressed. We extracted proteins from early embryos of various genotypes and detected Klar by Western analysis (Figure 2A). The Klar β null allele *klar^YG3 ^*[22] served as specificity control. Embryos from mothers carrying TM6B^Z ^over *Df(3L)emc^E12 ^*only have a single copy of the *klar *locus, and they express Klar β at reduced levels compared to the wild type. Klar^1 ^protein lacks the C-terminal 286 amino acids, and therefore migrates slightly faster than the wild-type protein [22]. Finally, Klar^MW ^was expressed at similar levels to the single copy of wild-type Klar^Z^. Its apparent molecular weight was similar to that of Klar^Z^, but larger than that of Klar^1^; these observations are consistent with a lack of ~60 amino acids in Klar^MW^.

To test if the mutant Klar was present on lipid droplets, we employed the assay previously used [22] to demonstrate that Klar is associated with embryonic lipid droplets: *in-vivo *centrifugation [11,35]. When syncytial embryos are centrifuged, the major organelles sort out by density. In particular, lipid droplets accumulate on the side of the embryo that points up during centrifugation. In the wild type, this lipid-droplet layer is highly enriched for Klar; mutant Klar proteins that fail to target to lipid droplets are absent from this layer [22]. We therefore determined Klar distribution in centrifuged embryos of various genotypes (Figure 2B). While in *klar^Z ^*Klar was highly enriched on the droplet layer, Klar signal was absent from the droplet layer in the LD truncation mutant *klar^MW^*. We conclude that truncation of the LD domain abolishes targeting of Klar to lipid droplets. Thus, the LD domain is indeed necessary for droplet localization *in vivo*.

### The LD domain truncation disrupts lipid-droplet transport in embryos

**Figure 3:**
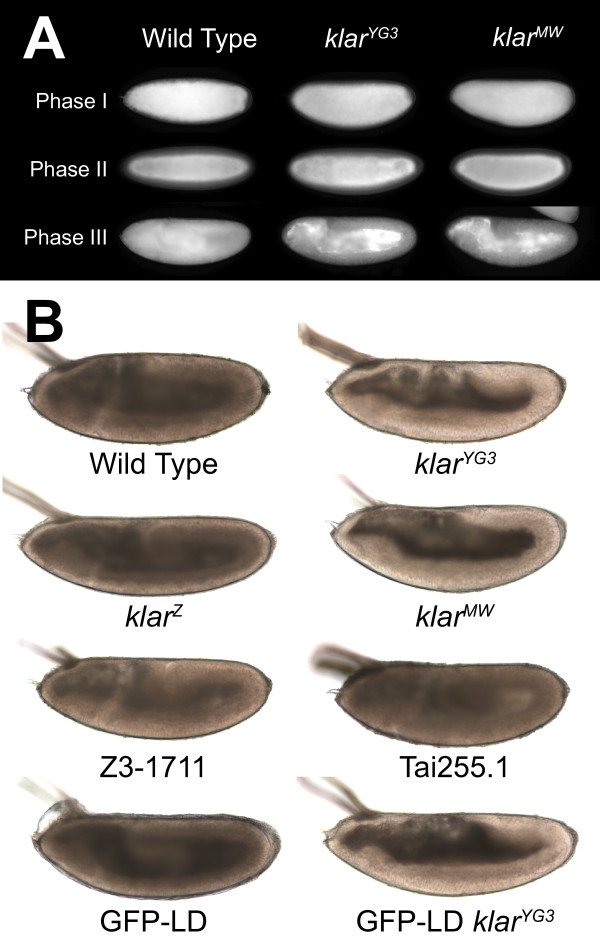
**The *klar^MW ^*allele does not support normal lipid-droplet transport in embryos**. (A) Embryos were fixed and stained with Nile Red to reveal lipid droplets. In all genotypes shown, lipid droplets are spread throughout the periphery in Phase I and move inward in Phase II. In the wild type, lipid droplets shift back into the periphery in Phase III. In *klar^YG3 ^*and *klar^MW^*, the bulk of lipid droplets remains in the central yolk in Phase III. (B) Living embryos were placed in halocarbon oil and imaged by bright-field microscopy after Phase III, as described [19,22]. Embryo transparency reveals global lipid-droplet distribution [19]. *Klar^YG3^*, *klar^MW^*, and GFP-LD *klar^YG3 ^*embryos display a transparent periphery, indicating that lipid droplets failed to spread out from their inward accumulation in Phase II. For all other genotypes shown, embryos are opaque, characteristic of normal outward droplet transport. Normal transport in the wild-type strain Tai255.1 has been observed previously [58].

During early embryogenesis, lipid droplets display stereotyped shifts in their overall distribution as the relative balance of plus- and minus-end motion changes in a temporally controlled manner [19]. In wild-type embryos, droplets are initially found throughout the periphery (Phase I: syncytial blastoderm). During cellularization (Phase II), lipid droplets move inwards, deplete from peripheral regions, and accumulate around the central yolk. This accumulation reverses during gastrulation (Phase III): the droplet population shifts outward and disperses throughout the embryo periphery. These shifts can be revealed by staining fixed embryos with the lipid-droplet specific dye Nile Red (Figure 3A) or by observing living embryos under transmitted light (Figure 3B): Since lipid droplets scatter light, cytoplasm full of droplets is opaque, while cytoplasm depleted of droplets is transparent [19]. As a result, wild-type embryos have a clear periphery in Phase II (not shown) and a cloudy periphery in Phase III (Figure 3B).

Klar β is important for the correct balance between plus- and minus-end motion. In the absence of Klar β, the global droplet distribution in Phases I and II is similar to the wild type, but becomes dramatically different in Phase III: lipid droplets remain around the center, instead of shifting back to the periphery. To assess if the LD domain contributes to Klar's function in this transport process, we compared *klar^MW ^*and *klar^Z ^*embryos. In both genotypes, the periphery turned clear in Phase II (not shown), just like for all other *klar *alleles previously characterized. In Phase III, *klar^Z ^*embryos were opaque, like the wild type; a single copy of *klar *is indeed sufficient to support normal net droplet transport in Phase III [19]. In contrast, *klar^MW ^*embryos were clear, very similar to embryos lacking Klar β altogether (Figure 3B). This droplet mislocalization was confirmed by Nile Red staining (Figure 3A): In *klar^MW ^*embryos, droplets accumulate around the central yolk in Phase II, and stay centrally in Phase III. Thus, *klar^MW ^*behaves like a Klar β loss-of-function allele. We conclude that the LD domain is essential for Klar β function. Since it also disrupts the localization of Klar β to droplets, we conclude that proper targeting to droplets is necessary for Klar β to control droplet motion. Presumably, Klar β has to be in close proximity to the motors it regulates.

### Sequence variation in the LD domain

The TILLING approach had uncovered a second strain (Z3-1770) with mutations in exon 15ext. We verified those changes by genomic sequencing; they are predicted to result in amino-acid changes at five positions (Figure 1A). In addition, in a survey of various laboratory strains, we found three amino-acid changes in Tai255.1, a stock collected from the wild in 1983. None of these changes apparently compromise Klar's function: In both strains, Klar localized to lipid droplets (data not shown), and lipid droplets displayed normal outward transport in Phase III (Figure 3B).

To identify other positions in the LD domain at which variation can be tolerated, we compared the sequence of this domain across seventeen fly species (Figure 1B). More than half of the LD domain displays dramatic sequence variation over this evolutionary time span (~250 Mya). But a 46 amino-acid stretch is highly conserved: 31 positions were either identical or similar among these species. This conservation suggests that this region is functionally important, likely for droplet targeting. This region is deleted in the non-targeting *klar^MW ^*allele (arrow in Figure 1B).

### The LD domain truncation supports nuclear migration in photoreceptors

Besides droplet transport, the best-characterized role of Klar is in the migration of nuclei in developing photoreceptors [19,24]. Here, Klar localizes to the nuclear envelope [24], and has been proposed to act as an anchor for cytoplasmic dynein [36]. Numerous lines of evidence suggest that this transport process requires the Klar α isoform [22,24], but the available data do not clearly address if - in addition to Klar α - Klar β is also required to support nuclear migration. This uncertainty is in large part due to the fact that previously it was not possible to selectively abolish just Klar β function without also affecting Klar α [22,27]. *Klar^MW ^*now provides a unique opportunity to address if Klar β is required for proper positioning of photoreceptor nuclei.

**Figure 4:**
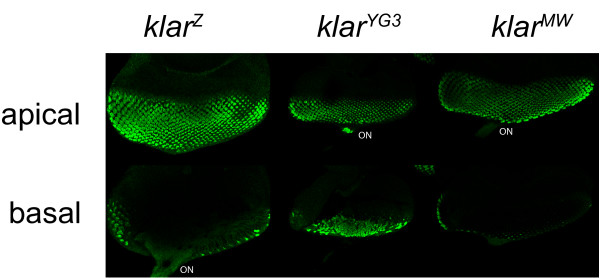
**The *klar^MW ^*allele supports nuclear migration in photoreceptors**. Eye discs from third-instar larvae were fixed, stained with anti-Elav to reveal photoreceptor nuclei and examined by confocal microscopy. In *klar^Z ^*discs, the photoreceptor nuclei are present in apical positions; a few nuclei are visible in the basal section because the disc curves at the edges. Nuclei are absent from the optical nerve (ON). This is the pattern observed in wild-type discs [22,24]. In *klar^YG3 ^*discs, many nuclei are found basally and in the optical nerve because apical migration of nuclei has failed. *Klar^MW ^*discs display the wild-type pattern.

To reveal the position of photoreceptor nuclei in third-instar eye discs, we employed the marker Elav (Figure 4). We then examined apical and basal focal planes of these discs for the presence of Elav-positive nuclei. In the wild type (not shown) and in *klar^Z^*, only the apical sections had nuclei, indicating successful migration of nuclei. In *klar^YG3 ^*(both Klar α and Klar β disrupted), nuclei were present in both apical and basal sections, indicating disrupted nuclear migration; nuclei were also found in the optical nerve. In *klar^MW ^*eye discs, nuclei were apical and not present basally nor in the optical nerve. Thus, Klar β is not essential for apical positioning of photoreceptor nuclei.

### A GFP-tagged LD domain localizes to embryonic lipid droplets

**Figure 5:**
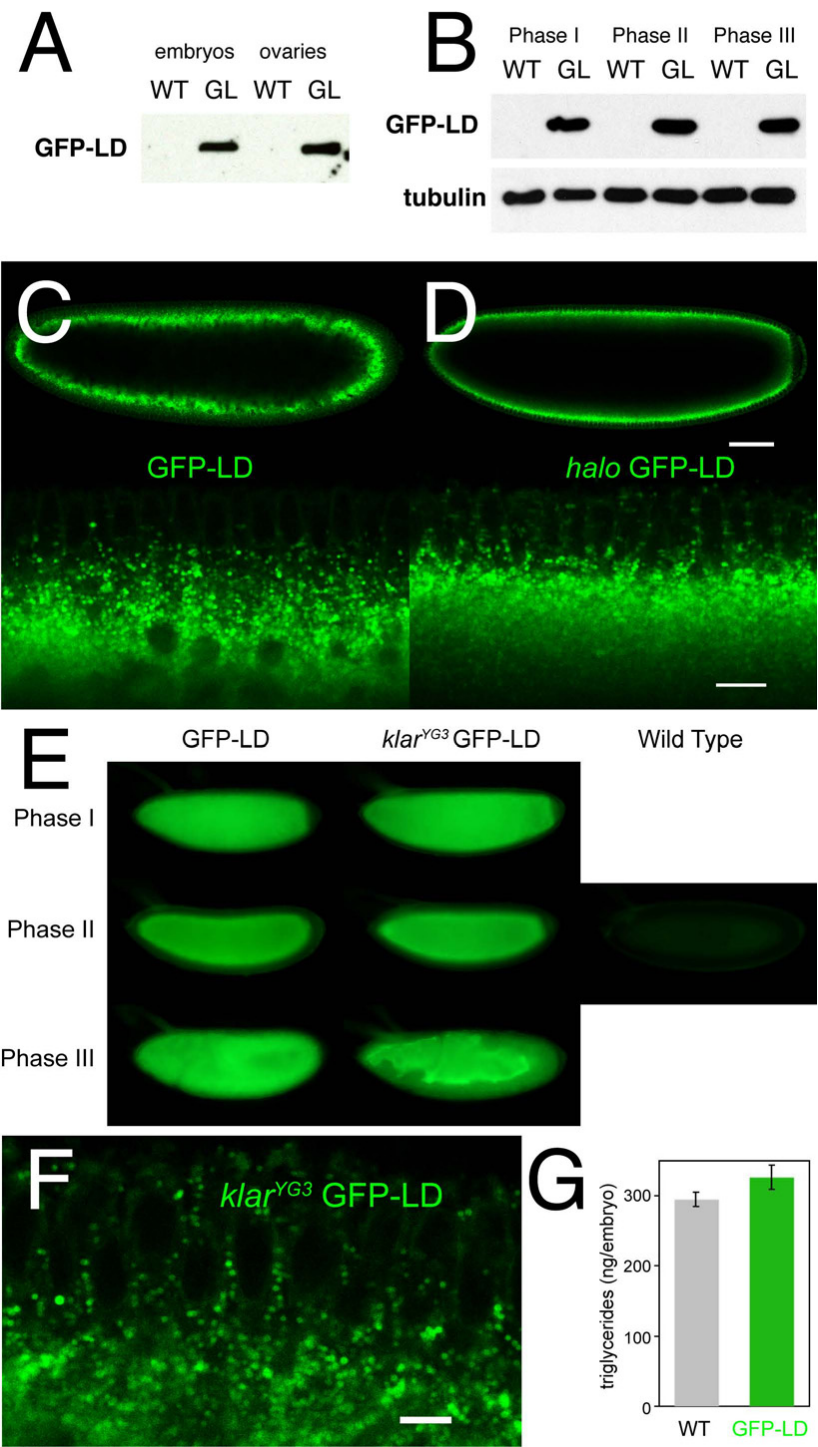
**GFP-LD distribution in early embryos**. (A, B) Western analysis of wild-type (WT) and GFP-LD expressing (GL) animals. GL samples are from females carrying both a GFP-LD transgene and the matα4-Gal4-VP16 driver or from embryos laid by these females. GFP-LD was detected with anti-GFP antibodies; corresponding WT samples demonstrate specificity of detection. (A) GFP-LD is present in both ovaries and embryos. (B) GFP-LD is expressed at similar levels in Phase I, II, and III embryos. Tubulin serves as loading control. (C, D) GFP-LD expressing embryos examined live by confocal microscopy. In otherwise wild-type embryos, GFP-LD is present in distinct puncta that accumulate around the central yolk (C). In embryos mutant for Halo, GFP-LD puncta accumulate just under the nuclei (D); these puncta are shifted outwards relative to the embryos in C. Top: whole-embryo view. Bottom: detail of the embryonic periphery. The embryos in C and D are age-matched (top: early Phase II; bottom: mid Phase II). Scale bars in D represent 60 μm (top) and 10 μm (bottom), respectively. (E) GFP-LD expressing embryos imaged live by epifluorescence microscopy. Comparison to a wild-type embryo demonstrates that most of the signal is due to GFP-LD. Global distribution of GFP-LD in Phases I, II, and III mimics the distribution of lipid droplets at these embryonic stages (Fig. 3A); in particular, GFP-LD is spread throughout the periphery in Phase III if endogenous Klar β is present, but not if it is absent. (F) Even when expressed in the absence of endogenous Klar β, GFP-LD is present in discrete puncta (scale bar = 6 μm). (G) Triglyceride levels in wild-type and GFP-LD expressing embryos (0-1.5 hrs old) are very similar. Error bars represent the standard deviation from two different experiments.

The previous analysis established that the LD domain is necessary *in vivo *for the droplet localization of Klar as well as for Klar's role in regulating droplet transport. To address whether the LD domain is sufficient for these functions, we generated transgenes to allow Gal4-mediated expression of a GFP-LD fusion protein. Since the lipid droplets of the early embryo originate during oogenesis, we combined these transgenes with Gal4 drivers specifically expressed in the female germ line (matα4-Gal4-VP16), so that the fusion protein could be produced when those lipid droplets are first generated. Western analysis confirmed that GFP-LD was expressed in ovaries and embryos (Figure 5A, B). Molecular weight standards indicate that its apparent molecular weight is in good agreement with the predicted molecular weight of 41.4 kDa (data not shown).

To determine the intracellular distribution of GFP-LD, we detected GFP fluorescence in living and in fixed embryos or stained fixed embryos with anti-GFP antibodies. All three methods revealed that the fusion protein was present in discrete puncta (Figure 5C, and data not shown), reminiscent of lipid droplets in size and abundance. These GFP-LD puncta moved actively, in a back-and forth manner (see the movies provided as Additional Files 1 and 2), similar to the bidirectional movement of lipid droplets [19]. In addition, the global distribution of GFP signal also mimicked that of lipid droplets (Figure 5E). The generally peripheral distribution in Phase I was followed by accumulation around the central yolk in Phase II. In Phase III, GFP-LD was again broadly peripheral.

If GFP-LD indeed marks lipid droplets, its distribution should change predictably if lipid-droplet transport is altered. Inward transport of lipid droplets in Phase II requires the transport regulator Halo; in its absence, lipid droplets accumulate close to the plasma membrane, under the nuclei, rather than around the yolk in the center [18]. Halo has no known role in the transport of any cargo beyond lipid droplets. In embryos lacking Halo, GFP-LD puncta also accumulate under the nuclei (Figure 5D), instead of around the yolk (Figure 5C). Furthermore, outward transport of lipid droplets in Phase III requires Klar β (Figure 3A). In the absence of endogenous Klar β, GFP-LD remained highly enriched in the yolk sack in Phase III (Figure 5E), just like lipid droplets [19,22]. Thus, in these genotypes, GFP-LD distribution reflects the distribution of lipid droplets.

**Figure 6:**
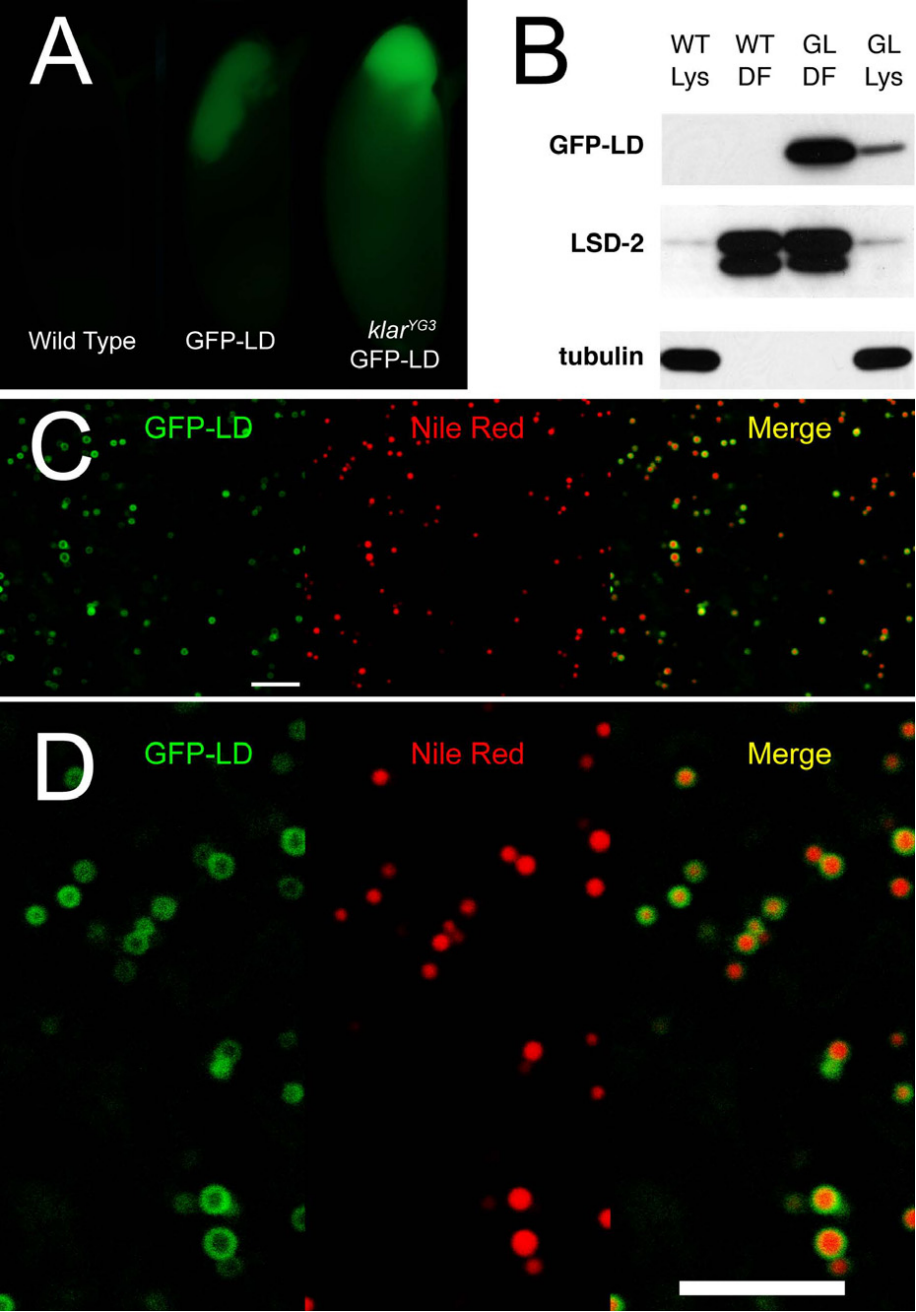
**GFP-LD puncta are lipid droplets**. (A, B) GFP-LD co-purifies with lipid droplets. (A) Pre-cellularization embryos were embedded in agar and centrifuged. In the image, the embryos are arranged such that the lipid-droplet layer points up. GFP-LD is highly enriched in the droplet layer, whether or not the embryos express endogenous Klar β. Under the same imaging conditions, autofluorescence in wild-type embryos is negligible. (B) Lipid droplets were isolated from wild-type (WT) and GFP-LD expressing (GL) embryos by floatation. Equal amounts of protein from embryo lysates (Lys) and from the droplet fraction (DF) were compared by Western analysis. The cytoplasmic protein tubulin is absent from the droplet fraction. Both the lipid-droplet protein LSD-2 and GFP-LD are highly enriched in the droplet fraction. GFP-LD was detected as in Fig. 5. (C, D) GFP-LD expressing embryos were fixed and stained with Nile Red to reveal lipid droplets. GFP-LD is present in rings around lipid droplets; intensity of GFP-LD signal varies between droplets (scale bar = 5 μm). Panel D shows a magnified view of parts of panel C.

Finally, at higher magnification, the GFP-LD structures appeared as rings (Figure 6C, D), in the known size-range of embryonic lipid droplets (~500 nm in diameter). Such ring structures are very characteristic for lipid droplets, as droplet proteins accumulate at the droplet surface and are excluded from the hydrophobic core full of neutral lipids [37]. Rings of GFP-LD were apparent in both fixed and living embryos. Co-staining of fixed embryos with the droplet-specific dye Nile Red revealed that GFP-LD signal was present around Nile-Red positive structures (Figure 6C, D). We conclude that the GFP-LD puncta are lipid droplets.

There are two unexpected patterns of the GFP-LD signal. First, it is present in fairly uniform rings, rather than the discrete spots observed for endogenous Klar β [22]. Full-length Klar β might be restricted to certain regions on the droplet surface via interactions of the N-terminal region with other proteins. Alternatively, the binding partners that keep Klar β locally restricted may be limiting, present in lower amounts than the well expressed GFP-LD. Second, only some of the Nile-Red positive structures display strong GFP-LD signal (Figure 6C, D), while on others GFP levels were much weaker or barely detectable. It is striking that drastic intensity variations can be observed between neighboring lipid droplets in the same cell. This observation suggests that GFP-LD displays differential affinity to different droplets or that, once droplets are generated with distinct GFP-LD levels, GFP-LD does not readily exchange between droplets.

### GFP-LD localizes to droplets in the absence of endogenous Klar β

The above analysis suggests that the LD domain is sufficient to target an unrelated protein to lipid droplets. This conclusion, however, might be misleading if the LD domain were a dimerization motif. In that case, GFP-LD could physically interact with full-length Klar β and would be targeted to lipid droplets secondarily, even if full length Klar were to bind to the droplets via other domains. Although the analysis of *klar^MW ^*makes this possibility unlikely, we performed a rigorous test of this idea by examining GFP-LD in a Klar β null background. GFP-LD was still present in distinct cytoplasmic rings the size of lipid droplets (Figure 5F) that moved bidirectionally (not shown). In addition, global GFP-LD distribution followed that of lipid droplets: throughout the periphery in Phase I, and accumulated in or around the central yolk in Phases II and III (Figure 5E). Thus, GFP-LD localization to lipid droplets does not require endogenous Klar β.

### The GFP-LD fusion co-purifies with lipid droplets

Lipid droplets are rich in neutral lipids and have a low buoyant density. This property makes it possible to biochemically separate droplets from other cellular structures. We therefore asked if GFP-LD copurifies with lipid droplets. In a first test, we employed *in-vivo *centrifugation of intact embryos [35]. In such centrifuged embryos, GFP-LD signal was highly enriched in the lipid-droplet layer (Figure 6A), just like endogenous Klar (Figure 2B). This enrichment is not due to the GFP portion as many other GFP fusion proteins are excluded from the droplet layer in this assay [11]. It also does not depend on endogenous Klar β since GFP-LD was enriched in the droplet layer in the Klar β null background (Figure 6A). Second, we lysed embryos and enriched for droplets using a sucrose step-gradient (Figure 6B). The top, lipid-droplet fraction was highly depleted for the cytoplasmic protein tubulin and greatly enriched for the *bona-fide *lipid-droplet protein LSD-2. GFP-LD was similarly enriched in this fraction. Taken together, these two approaches demonstrate that GFP-LD co-purifies with lipid droplets and provide independent evidence that the LD domain is sufficient to target an unrelated protein to lipid droplets.

### The LD domain mediates droplet localization in many cell types

How proteins are targeted to lipid droplets is not well understood [1]. Droplet-localized proteins fall into two broad classes. Some proteins localize to lipid droplets in essentially all cells they are expressed in, such as the Perilipin family members PLIN1and PLIN2 (formerly called Perilipin and ADRP, respectively) [21,38]. This constitutive localization is in contrast to the conditional recruitment of, *e.g*., hormone-sensitive lipase; this enzyme moves from a general cytoplasmic distribution to the surface of lipid droplets in response to hormonal signaling [39]. In addition, specific proteins from other cellular compartments localize to lipid droplets only in certain developmental stages or under specific environmental conditions [40]. For early embyronic droplets in *Drosophila*, certain histones are such conditional lipid-droplet proteins [11]; many more proteins potentially behave similarly as the proteomes of embryonic and of fat-body droplets show considerable differences [11,41]. We therefore asked whether localization of GFP-LD occurs only in early embryos or is a general phenomenon.

**Figure 7:**
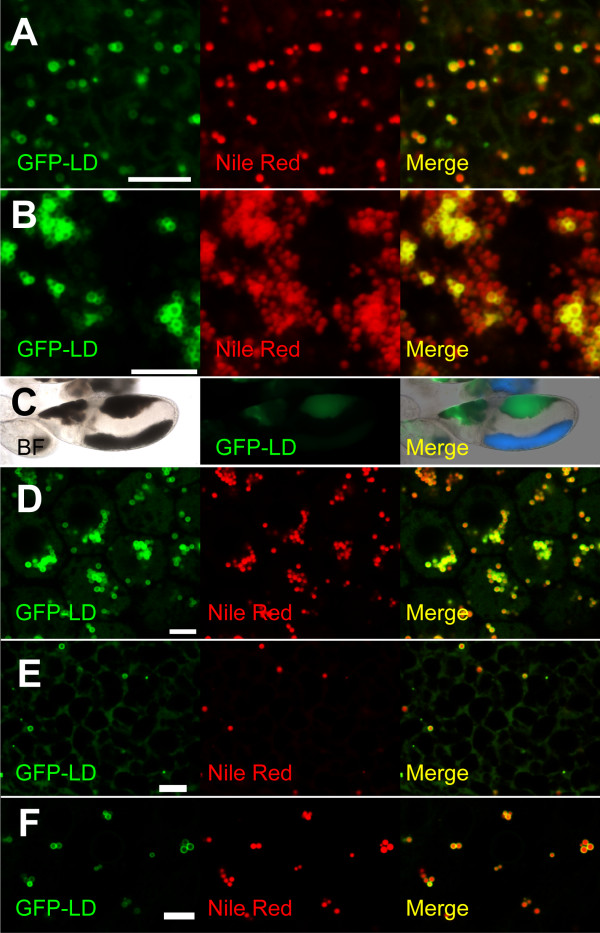
**GFP-LD fusion protein localizes to lipid droplets in many types of cells**. The intracellular distribution of GFP-LD was examined in animals expressing GFP-LD in the female germ line (A, B, C: matα4-Gal4-VP16 driver) or in somatic cells (D, E, F: Act5C-Gal4 driver). Colocalization with Nile Red signal (A, B, D, E, F) or enrichment in the lipid-droplet layer after centrifugation (C) indicates that GFP-LD is associated with lipid droplets. (A) Stage 13 oocyte. (B) Stage 10 nurse cells. (C) Egg chamber after centrifugation. Bright-field microscopy (BF) reveals distinct layering. Prominent brown lipid-droplet layers are evident in the nurse cells (left) and the oocyte (right); the oocyte also shows a gray yolk layer. GFP-LD is enriched in the droplet layers of both nurse cells and oocyte. The merged image also shows yolk autofluoresence in blue. (D) Follicle cells. (E) Larval salivary gland. (F) Wing imaginal disc. In all cases, the intensity of GFP-LD signal varies dramatically between lipid droplets. GFP-LD signal was also present, at lower levels, elsewhere in the cell (*e.g*., E). Scale bars = 5 μm.

We first examined ovaries since lipid droplets are abundant in nurse cells and in oocytes from mid-oogenesis onwards. GFP-LD expressed with a Gal4 driver specific for the female germ-line (matα4-Gal4-VP16) accumulates during oogenesis (Figure 5A), and is present in ring structures in both oocytes and nurse cells (Figure 7A, B). These rings co-stained with Nile Red and are apparently of low buoyant density: In centrifuged ovaries, where the major constituents of nurse cells and oocytes sort out by density [11], GFP-LD was highly enriched in the lipid-droplet layer (Figure 7C). We conclude that GFP-LD is present on lipid droplets in oocytes and nurse cells.

Using the ubiquitously active Act5C-Gal4 driver, we also expressed GFP-LD in somatic cells. In a number of adult and larval tissues, we found discrete GFP puncta, including in follicle cells in the adult ovary, larval salivary glands, and imaginal discs. These structures are lipid droplets, as they appear as rings at higher magnification and co-label with Nile Red (Figure 7D, E, F). Since in the wild type lipid droplets are abundant in certain imaginal discs [42], in follicle cells (MAW, unpublished observations), as well as in nurse cells and oocytes [43], GFP-LD likely does not induce lipid storage *de-novo *in these tissues, but localizes to pre-existing droplets.

In summary, targeting of the LD domain to lipid droplets is not an embryo-specific phenomenon. GFP-LD also localizes to lipid droplets in the female germ line and in a number of somatic tissues. In addition, an analogous RFP-LD fusion protein localizes to lipid droplets in cultured *Drosophila *cells [22]. Thus, GFP-LD is not a conditional droplet protein, and the mechanism targeting the LD domain to lipid droplets is quite general.

## Discussion

### A *klar *allele specific for the Klar β isoform

Because the *klar *locus encodes at least three proteins with different exon content, different lesions in *klar *have distinct effects on the isoforms and thus on distinct biological processes [22]. Lesions in exons 0 through 15 disrupt both Klar α and Klar β; these so-called class I alleles impair both nuclear migration in photoreceptors and motion of embryonic lipid droplets. Lesions in exons 16 through 18 disrupt Klar α and Klar γ; such class II alleles impair nuclear migration, but not droplet motion. The new allele *klar^MW ^*disrupts Klar β but not Klar α; it alters droplet motion but not nuclear migration. It constitutes a new type of allele (class III) that selectively impairs the β isoform.

*Klar^MW ^*provides a unique tool to separately examine the functions of Klar α and Klar β. Klar is widely expressed [22] and has been implicated in a number of biological processes beyond droplet motion and nuclear migration [23,44-48]. In most cases, it is unknown which Klar isoform is involved. The combination of class I, II and III alleles should make it possible to disentangle this functional complexity.

### Droplet targeting by the LD domain

Our analysis of GFP-LD fusions suggests that targeting of Klar β to lipid droplets is a multi-step process. In many cells, GFP-LD strongly accumulates on lipid droplets (Figure 6, 7). In mid-stage oocytes, in follicle cells, and in the salivary gland, GFP fluorescence is also detected diffusely and in membranous structures and tubules reminiscent of the nuclear envelope or the ER (not shown). Under sensitive imaging conditions, similar additional GFP-LD distribution becomes apparent also in early embryos. These observations suggest that - in addition to strong affinity to lipid droplets - GFP-LD has a weak affinity to certain membranous compartments. We do not know yet whether this additional localization represents nascent GFP-LD in transition to lipid droplets, ectopic localization due to high levels of expression, or genuine multiple targeting. Dual localization of proteins to both the ER and lipid droplets is common, presumably because lipid droplets originate from the ER [1].

Nevertheless, GFP-LD clearly accumulates strongly on lipid droplets in many tissues, forming characteristic ring structures. Intriguingly, this distribution is distinct from the distribution of full-length Klar β, which is present in discrete dots on embryonic droplets [22], a distribution similar to that of the motor dynein [14]; thus, full-length Klar may be recruited to distinct spots by physical interactions with microtubule motors. GFP-LD may not be restricted to such dots because interactions with the motors might require sequences in the N-terminal region of Klar β or because GFP-LD is overexpressed relative to its interaction partners. We favor the former explanation since even those lipid droplets with comparatively low GFP-LD signal show a ring-like distribution of GFP-LD (Figure 6C, D). In the future, these hypotheses can be distinguished by comparing the distribution of GFP-LD and GFP-Klar β expressed at similar levels.

What is the molecular mechanism by which the LD domain, a region just 114 amino acids in length, is recruited to lipid droplets? Evolutionary conservation and mutational analysis point to a 46 amino acid region as critical for targeting (Figure 1B). Many proteins localize to droplets via hydrophobic targeting motifs thought to insert into the phospholipid monolayer surrounding droplets and to make contacts with the hydrophobic core. Examples include the proline knot motif of plant oleosins [49], hydrophobic patches in caveolin [50] or amphipathic helices in various Perilipin family members [21]. Consistent with this possibility, the 46 aa conserved region of the LD domain is fairly hydrophobic (light blue residues in Figure 1B) and the regular interspersion of charged residues is compatible with this region forming an amphipathic helix (Figure 1C). Alternatively, this motif may allow the LD domain to physically interact with resident droplet proteins, *e.g*. just like hormone-sensitive lipase is recruited to droplets by binding to PLIN1 [39]. If so, the binding partners of the LD domain cannot be exclusive to embyronic droplets since droplet targeting occurs in many types of cells (Figure 7). As a first step towards uncovering the targeting mechanism, a structure-function analysis of this region should reveal which features of the sequence (*e.g*., general hydrophobicity versus specific residues) are essential for proper droplet targeting. These studies can be conducted in the more accessible cultured-cell system, since the LD domain shows the same targeting properties *in vivo *(Figure 6, 7) as in cultured cells [22].

Since GFP-LD localizes to the droplet surface, it might potentially interfere with lipid metabolism. For example, in cultured mammalian cells, overexpression of PLIN2 promotes lipid-droplet accumulation, presumably by shielding the droplets from access by lipases [51,52]. We have not noticed dramatic effects on lipid storage upon GFP-LD overexpression. Total triglyceride levels in wild-type and GFP-LD expressing embryos are very similar (Figure 5G), and in a range of cell types, lipid droplets carrying high levels of GFP-LD are very similar in size to nearby droplets with low GFP-LD levels (Figure 6 and 7). These data do not rule out that GFP-LD causes minor quantitative changes in lipid storage or only produces effects in specific cell types or under certain physiological conditions.

### Why is GFP-LD distribution not uniform?

It is striking that not all lipid droplets accumulate GFP-LD to the same level. Lipid droplets in close proximity in the same cell can have dramatically different GFP-LD signal (Figures 6, 7). An exciting development in recent years has been the realization that not all lipid droplets within a given cell are identical; in particular, they can carry different proteins [37,38,41,53]. However, the lipid droplets in early *Drosophila *embryos appear quite uniform, in size distribution and motile behavior [14,18,19]; droplet proteins previously examined displayed no obvious variation between droplets [11,20]. To our knowledge, the uneven distribution of GFP-LD is the first hint that different embyronic droplets might have distinct properties.

Currently, we cannot distinguish whether GFP-LD is preferentially recruited to certain types of droplets existing naturally or whether GFP-LD expression causes differences between droplets. For example, Gal4 drivers often show mosaic expression in nurse cells [54]; and we sometimes observed, in the same egg chamber, nurse cells with variable GFP-LD expression (data not shown). Droplets that originated in different nurse cells may therefore carry distinct levels of GFP-LD once they reach the oocyte. We suspect that such mosaic expression does not fully account for the differential labeling because we observe drastic variation in GFP-LD levels also between droplets in single nurse cells as well as in other cells (Figure 7).

### The role of the LD domain for droplet transport

The LD domain is necessary not only for droplet localization of Klar β, but also for Klar β's function in regulating droplet transport. Our results indicate that although the LD domain is sufficient for droplet localization, by itself it does not mediate Klar's transport functions. When expressed in the Klar β null background, most GFP-LD signal remains in the yolk sack in Phase III (Figure 5E). In addition, Klar β null embryos remain clear in Phase III, whether or not they express GFP-LD (Figure 3B). Simply targeting GFP-LD to lipid droplets is apparently not sufficient to restore Klar β's function.

Although it is conceivable that GFP-LD expression levels were simply not high enough for rescue of the transport defects, we disfavor this explanation since a full-length, GFP-tagged Klar β construct expressed at much lower levels is sufficient to profoundly alter droplet motion (YVY and MAW, unpublished observations). We therefore conclude that proper regulation of droplet motors requires the N-terminal region of Klar β and not just the LD domain. This model is further supported by the fact that Klar α shares those N-terminal regions and is also involved in motor regulation.

Whether the LD domain simply targets Klar β to lipid droplets or has additional functions is not yet clear. For example, because in yeast the LD domain can interact with the droplet protein LSD-2, it has been suggested that it participates in transmitting developmental signals from LSD-2 via Klar to motors [20]. In the future, it will be interesting to investigate whether expression of GFP-LD alters subtle aspects of droplet motion, *e.g*., the exact distances traveled in a single run or the frequency of pausing [14].

However, our data suggest that such effects, if they exist at all, do not alter the net outcome of transport. GFP-LD expressing embryos displayed the same transparency changes as the wild type: the embryo periphery became transparent in Phase II and cloudy in Phase III (Figure 3B, and data not shown). Also, in the presence of GFP-LD, inward droplet transport in Phase II still depends on Halo (Figure 5D) and outward droplet transport in Phase III depends on endogenous Klar β (Figure 5E), just as for embryos not expressing the fusion protein [18]. These conclusions hold true whether we examine transparency changes in the embryos (to reveal the behavior of the entire droplet population) or GFP fluorescence directly (Figure 3B; Figure 5D, E).

Since LD has the ability to bind to lipid droplets in a wide range of cells, Klar β may control droplet motility in many tissues. In *Drosophila*, droplet motion has so far been described only in early embryos [19] and in oocytes [55], but no systematic analysis has been conducted. New GFP fusions to mark lipid droplets *in vivo *[37,56], including the GFP-LD constructs described here, will make it possible to address to what extent droplets move in other tissues and whether disruption of Klar β alters that movement.

## Conclusions

To test the function of the LD domain of Klar β, we generated inducible GFP-LD fusion constructs and identified a *klar *allele that specifically disrupts the LD domain. Using these tools, we demonstrate that the LD domain is both necessary and sufficient for droplet targeting *in vivo*. We conclude that Klar β is targeted to lipid droplets via an isoform-specific protein motif, just like Klar α is targeted to the nuclear envelope via the KASH domain. Thus, it is controlled inclusion of cis-acting targeting sequences that mediates the differential intracellular localization of Klar in distinct tissues. Although Klar's LD domain is necessary for Klar β to act in the regulation of lipid-droplet transport, by itself it does not mediate Klar's transport functions. Likely it is the N-terminal regions shared between Klar α and Klar β that mediate motor regulation. In this model, variable C-terminal targeting sequences control Klar's intracellular distribution and thus dictate which subset of intracellular motors is controlled by Klar.

## Methods

### Fly stocks

Oregon R was used as the wild-type stock. The stocks carrying *klar *alleles *klar^YG3 ^*and *klar^1 ^*and the deficiency *Df(3L)emc^E12 ^*were described previously [19,22]. The collection of mutagenized third chromosomes was generated by the Zuker laboratory [31]. Line Z3-1711 might represent a different chromosome than the others from the collection: it did not display the recessive eye color markers characteristic for these stocks [31], and it had five simultaneous amino-acid changes in the LD domain (Figure 1A). However, for the purposes of the analysis described here, the exact origin of this chromosome is not important. The critical information is the presence of these mutations in the LD domain and the wild-type phenotype for droplet transport and Klar localization. Tai255.1 is a wild-type *D. melanogaster *strain isolated in 1983 at Ivory Coast [57]; it displays normal net droplet transport [58].

To generate the GFP-LD expressing flies, GFP was amplified from pEGP-C1 (Clontech) and cloned into the *Kpn*I and *Not*I sites of pUASp [54]. A tobacco etch virus (TEV) protease site (GAGAATTTGTATTTTCAGGGT) was generated by oligo nucleotide synthesis and cloned 3' to the GFP gene. The LD domain was amplified from cDNA clone LD08331 [22] and cloned in frame 3' to the TEV site. The resulting plasmid was injected into *Drosophila *embryos by Genetic Services, Inc. (Cambridge, MA). Transgenic flies were selected by eye color. In total, twenty lines mapping to the X, second or third chromosome were established. To express the fusion proteins, transgenic animals were crossed with lines carrying matβ4-Gal4-VP16 or Act5C-Gal4 drivers.

### TILLING, genomic sequencing, and sequence analysis

TILLING analysis was performed by the Fly-TILL service as described [32]. In ~6000 strains from the Zuker collection [31], we screened a 1519 bp genomic region from the end of exon 12 through the coding region of exon 15ext. Nucleotide changes were uncovered in 23 lines; in two cases (Z3-1711 and Z3-3772), these changes mapped to exon 15ext. For these two strains, we sequenced PCR products encompassing exon 15ext as described below. The nucleotide changes observed in line Z3-3772 were due to changes on the balancer chromosome (see main text for details).

DNA primers were created to bookend individual exons of *klar *piecemeal using annealing temperatures and GC content to determine the most effective oligo sequences (primer sequences available upon request). Purified DNA from adult flies was used to individually PCR amplify these genomic fragments. PCR products were depleted of free nucleotides with ExoSAP-IT and sequenced at the Life Sciences Core Laboratories Center at Cornell University. Sequencing results were compared to the canonical sequences available on FlyBase.

To determine the pattern of evolutionary conservation in the LD domain, we first identified the likely *klar *exon 15 in the available genome annotations for the *Drosophila *and mosquito species accessible via the FlyBase BLAST server. We also identified related sequences from the *G. morsitans *genome project (Sanger Institute) as well as in a cDNA from the medfly *C. capitata *(GenBank # FG077614.1). In *Drosophila melanogaster*, exon 15ext follows immediately downstream of exon 15. In all 16 additional fly species examined, the corresponding DNA sequences downstream of exon 15 have the potential to encode proteins with significant similarity to the *D. melanogaster *LD domain (Figure 1B). These protein sequences were aligned with Clustal2.

The helical wheel in Figure 1C was drawn using the helical wheel plotting script from the Zidovetzki laboratory, UC Riverside http://rzlab.ucr.edu/scripts/wheel/wheel.cgi.

### *In-vivo *centrifugation, fixation, immunostaining, and imaging

Living embryos were centrifuged to separate lipid droplets from other cellular components, as described [35]. Embryos were either embedded in agar to keep them in a fixed orientation during centrifugation, or they were centrifuged in random orientations in microcentrifuge tubes filled with buffer, and the lipid-droplet layer was identified by its characteristic appearance by bright-field microscopy. To separate organelles by density in oocytes and nurse cells, females were centrifuged in buffer-filled microfuge tubes as described [11].

To stain lipid droplets, dechorionated embryos or dissected fly tissues were fixed in 4% formaldehyde in PBS for 10-15 min using standard procedures [59,60]. For embryos, this treatment is sufficient to make the vitelline membrane permeable to Nile Red. After washing, these samples were suspended with 1% BSA in PBS and stained with Nile Red at 20 μg/ml. In some cases (Figure 7D, E, F), unfixed tissues were directly stained with Nile Red. For immunodetection of GFP or Klar, dechorionated embryos were heat fixed and devitellinized using standard heptane-methanol procedures [59,60]. They were stained either with mouse monoclonal Klar-M as described [22] or with rabbit anti-GFP (Torrey Pines Biolabs) at 1:10,000. Before use, the anti-GFP antibody was exposed to heat-fixed wild-type embryos to remove cross-reacting antibodies.

Two approaches were employed to examine GFP-LD fluorescence in living embryos. For confocal microscopy, embryos were hand-dechorionated, placed in halocarbon oil on a glass slide, and covered with a cover glass that was supported by spacers. For epifluorescence microscopy, embryos were placed into halocarbon oil on a glass slide. The halocarbon oil turns the chorion transparent (see Figure 3B).

Micrographs were acquired on a Leica SP5 confocal microscope or a Nikon Eclipse E600 fluorescence microscope with a 4MP Spot Insight camera. Images were processed in Adobe Photoshop and assembled with Adobe Illustrator.

### Western analysis

For Western analysis, proteins were typically separated on a 10% SDS PAGE gel and transferred to PVDF membranes using standard Towbin or CAPS transfer. Mouse anti- alpha tubulin (Sigma-Aldrich) was used at 1:10,000, and mouse anti-GFP (Roche) at 1:1000. LSD-2 was detected with a rabbit polyclonal anti-serum [20] at 1:20,000. To consistently detect Klar β, this general procedure was optimized: Proteins were separated on 6% gels, and transferred in 25 mM Tris, 192 mM glycine, 10% methanol, 0.01% SDS to PVDF membranes (2 hrs at 100 V). Membranes were sequentially exposed to Klar-M (1:50, overnight at 4°C), rat anti-mouse IgG (1:1000; 1 hr at room temperature) and HRP-conjugated goat anti-rabbit IgG (1:1000; 1 hr at room temperature).

### Droplet purification

To generate samples enriched in lipid droplets, we adapted the protocol described previously [11]. For each genotype, 150 μl of embryos (0-3 hrs old) were dechorionated, resuspended in 300 μl TKM (50 mM Tris, pH 7.4, 25 mM KCl, 5 mM MgCl_2_) containing 1 M sucrose plus protease inhibitor cocktail (Sigma-Aldrich), and then mechanically disrupted on ice. The lysate was transferred to a fresh 1.5 ml tube and overlaid sequentially with 200 μl TKM containing 0.5 M sucrose, 200 μl TKM containing 0.25 M sucrose, and 400 μl TKM. After centrifugation at 1000 g for 5 min, 5000 g for 5 min and 13400 g for 10 min, the buoyant lipid droplets were collected from the top of the gradient. The amount of protein in the isolated lipid droplets was measured by Bradford protein assay (Quick Start, Bio-Rad) before solubilizing the lipid droplets in SDS-containing buffer for subsequent SDS-PAGE analysis. Fractions such prepared are similar to those described by Cermelli et al. [11]: the overall pattern of major Coomassie-stainable proteins is similar; the droplet protein LSD-2 and several histones are consistently enriched, and the cytoplasmic protein tubulin is absent (Figure 6B; Li and Welte, unpublished observations).

### Triglyceride measurements

Embryos were collected for 1.5 hr, dechorionated with 50% bleach, and resuspended in triton salt solution [59,60]. 200 embryos before cellularization stages were handselected, resuspended in 200 μl homogenizing buffer (0.01 M KH_2_PO_4_, 1 mM EDTA, pH 7.4), and mechanically disrupted. 40 μl of this lysate were mixed with 1 ml activated Triglyceride Reagent (Liquicolor Triglycerides, Stanbio), and triglyceride levels were determined according to the manufacturer's instructions. For each experiment, three independent samples were analyzed per genotype, and the data shown in Figure 5G are based on two independent experiments.

## Authors' contributions

YVY generated and characterized the GFP-LD transgenes, contributed to the functional analysis of *klar^MW^*, consulted on a range of technical issues, and helped to draft the manuscript. ZL performed the biochemical purification of lipid droplets and the triglyceride measurements. NPR performed Western analysis, immunostaining, and genomic sequencing. JE performed the bulk of genomic sequencing, including primer design. MAW conceived and designed the study, performed the imaging analysis, aligned sequences and drafted the manuscript. All authors read and approved the final manuscript.

## Supplementary Material

Additional file 1 **Motion of GFP-LD puncta in living embryos**. Images were acquired by confocal microscopy at 2.5 images per second. Playback at 12 frames per second. Scale bar = 7.5 μm. Click here for file

Additional file 2 **Motion of GFP-LD puncta in living embryos**. Images were acquired by confocal microscopy at 1.35 images per second. Playback at 6 frames per second. Scale bar = 7.5 μm. Click here for file
